# Differences in left ventricular myocardial function and infarct size in female patients with ST elevation myocardial infarction and spontaneous coronary artery dissection

**DOI:** 10.3389/fcvm.2023.1280605

**Published:** 2024-01-08

**Authors:** Gordana Krljanac, Svetlana Apostolović, Marija Polovina, Ružica Maksimović, Olga Nedeljković Arsenović, Nemanja Đorđevic, Stefan Stanković, Lidija Savić, Ana Ušćumlić, Sanja Stanković, Milika Ašanin

**Affiliations:** ^1^ Cardiology Clinic, University Clinical Center of Serbia, Belgrade, Serbia; ^2^ Faculty of Medicine, University of Belgrade, Belgrade, Serbia; ^3^ Coronary Care Unit, Cardiology Clinic, University Clinical Center of Nis, Nis, Serbia; ^4^ Faculty of Medicine, University of Nis, Nis, Serbia; ^5^ Center for Radiology and Magnetic Resonance Imaging, University Clinical Center of Serbia, Belgrade, Serbia; ^6^ Center for Medical Biochemistry, University Clinical Center of Serbia, Belgrade, Serbia; ^7^ Faculty of Medical Sciences, University of Kragujevac, Kragujevac, Serbia

**Keywords:** spontaneous coronary artery dissection, myocardial infarction with ST-elevation, myocardial function, myocardial infarct size, echocardiography

## Abstract

**Introduction:**

Differences in pathophysiology, clinical presentation, and natural course of ST-elevation myocardial infarction in female patients due to either spontaneous dissection (SCAD-STEMI) or atherothrombotic occlusion (type 1 STEMI) have been discussed. Current knowledge on differences in left ventricular myocardial function and infarct size is limited. The aim of this study was to assess baseline clinical characteristics, imaging findings, and therapeutic approach and to compare differences in echocardiographic findings at baseline and 3-month follow-up in patients with SCAD-STEMI and type 1 STEMI.

**Methods:**

This was a prospective multicenter study of 32 female patients (18–55 years of age) presenting with either SCAD-STEMI due to left anterior descending coronary artery (LAD) dissection or type 1 STEMI due to atherothrombotic LAD occlusion.

**Results:**

The two groups were similar in age, risk factors, comorbidities, and complications. SCAD-STEMI patients more often had Thrombolysis in Myocardial Infarction 3 flow, while type 1 STEMI patients were more often treated with percutaneous coronary intervention and dual antiplatelet therapy. Baseline mean left ventricular (LV) ejection fraction (LVEF) was similar in the two groups (48.0% vs. 48.6%, *p* = 0.881), but there was a significant difference at the 3-month follow-up, driven by an improvement in LVEF in SCAD-STEMI compared to type 1 STEMI patients (Δ LVEF 10.1 ± 5.3% vs. 1.8 ± 5.1%, *p* = 0.002). LV global longitudinal strain was slightly improved in both groups at follow-up; however, the improvement was not significantly different between groups (−4.6 ± 2.9% vs. −2.0 ± 2.8%, *p* = 0.055).

**Conclusions:**

The results suggest that female patients with SCAD-STEMI are more likely to experience improvement in LV systolic function than type 1 STEMI patients.

## 1 Introduction

Distinct pathophysiological mechanisms underlying the development of type 1 ST-elevation myocardial infarction (STEMI) and myocardial infarction occurring due to spontaneous coronary artery dissection (SCAD), SCAD-STEMI, may be responsible for the differences in left ventricular (LV) function and myocardial infarct size in these two types of conditions (1, 2). Previous research findings suggested significant differences in the pathophysiology, clinical presentation, and natural course in female patients with ST-elevation myocardial infarction due to either spontaneous dissection (SCAD-STEMI) or atherothrombotic occlusion (type 1 STEMI) (3, 4).

Whether a more balanced process of infarct development in SCAD-STEMI could potentially result in smaller infarct sizes than the typical type 1 STEMI remains uncertain. It is important to note that the formation of myocardial infarction is a complex process. Contributing factors to infarct size include the type of infarct-related artery, severity and extent of coronary artery disease, location of the occlusion, the time it takes to restore blood flow by one of the revascularization procedures, and the overall health of the patient. In the case of SCAD-STEMI, various mechanisms may influence the infarct size, including the extent of the dissection, the occurrence of coronary artery healing, the presence of collateral blood flow, and the timing of coronary flow restoration (5). It is worth noting that most patients who present with SCAD typically have either small infarctions or no infarctions at all, and they also tend to have a preserved ejection fraction. However, those patients presenting as STEMI, Thrombolysis in Myocardial Infarction (TIMI) 0/1 flow at angiography, and/or multivessel SCAD are more likely to present with larger infarctions (5).

The aim of this research was to present baseline characteristics, risk factors, clinical findings, complications, laboratory analyses, and therapeutic approach and to compare differences in echocardiographic findings at baseline and 3-month follow-up in patients with SCAD-STEMI and type 1 STEMI.

## 2 Materials and methods

The study was conducted in 2023 at the University Clinical Centers in Belgrade and Nis, Serbia, as a prospective multicenter study. We included 32 consecutive adult female patients aged 18–55 years, presenting with either anterior SCAD-STEMI due to left anterior decedent coronary artery (LAD) dissection or type 1 STEMI due to atherothrombotic LAD occlusion. The patients were included prospectively between January 2023 and September 2023. The classification between SCAD-STEMI and type 1 STEMI was based on the findings of an emergency coronary angiography, which was performed at admission in all patients.

Patients who are either younger than 18 or older than 55 years; had a history of acute myocardial infarction (AMI) or coronary interventions, heart failure, uncontrolled hypertension, malignant diseases, obstructive pulmonary disease, hepatic or renal failure (eGFR ≤ 60 ml/min/1.73 m^2^), acute or chronic infections, ketoacidosis; or were treated with corticosteroids or immunosuppressive agents were excluded from the study. All patients were referred for coronary angiography immediately after admission. All type 1 STEMI female patients were treated with percutaneous coronary interventions (PCIs) with successfully establishing TIMI 3 flow after intervention. Patients with SCAD-STEMI were treated with optimal medical therapy in accordance with recommendations in previous studies (6) except in the setting of SCAD type 4, active/ongoing ischemia, and hemodynamic instability. Upon admission, a complete medical history was obtained and a physical exam with anthropometric measurements was performed. Blood samples were taken for laboratory analysis during hospitalization. Comprehensive echocardiographic exams were performed by experienced cardiologists at baseline and after 3-month follow-up and compared between groups. Cardiac magnetic resonance (CMR) was performed in clinically and therapeutically disputed cases. A clinical 1.5-T scanner (Siemens Avanto) was used to perform CMR imaging. The imaging protocols were standardized and unified (University Clinical Center of Serbia, Center of CMR). The standard protocol for morphological and functional assessment was followed, which included late gadolinium enhancement (LGE), T1, and T2 mapping using the MOLLI sequence before and after contrast medium application. Myocardial T1 and T2 mapping was performed in long-axis directions and three short-axis slices (base level, midventricular, and apex level) using a validated variant of a modified Look-Locker Imaging sequence (University Clinical Center of Serbia, Center of CMR, MOLLI). Late gadolinium enhancement imaging was performed 10 min after the administration of 0.1 mmol/kg of body weight of gadobutrol (Gadovist; Bayer). The interpretation of LGE images followed standardized post-processing recommendations by two observers based on the presence and predominant pattern as ischemic or non-ischemic. The mean time for performance of CMR was 15 ± 7 days. There was no significant difference in the mean time from SCAD-STEMI/type 1 STEMI infarct onset to CMR performance.

All standard echocardiographic examinations were performed using Vivid E95 (General Electric). Data were acquired with a 3.5-MHz transducer in the parasternal (long- and short-axis views) and apical (four- and two-chamber and apical long-axis views) views, utilizing echocardiographic methods such as M-mode, 2D, color Doppler, pulse Doppler, continuous Doppler, tissue Doppler, and speckle-tracking imaging. All measurements and definitions were in accordance with the guidelines of the European and American Society of Echocardiography (7, 8).

Two-dimensional speckle-tracking echocardiography (2D-STE) is a non-invasive ultrasound imaging technique that allows for an objective and quantitative evaluation of global and regional myocardial deformation. It is also used to assess left ventricular (LV) systolic and diastolic myocardial function. The recordings were performed with a frame rate between 50 and 70 frames/s and analyzed offline using General Electric software (EchoPAC software version 203 GE Medical Systems). All parameters of myocardial longitudinal strain were calculated offline in accordance with recommendation (9), and the global longitudinal strain (GLS) was analyzed on the 18-segment segmentation model.

**Figure 1:**
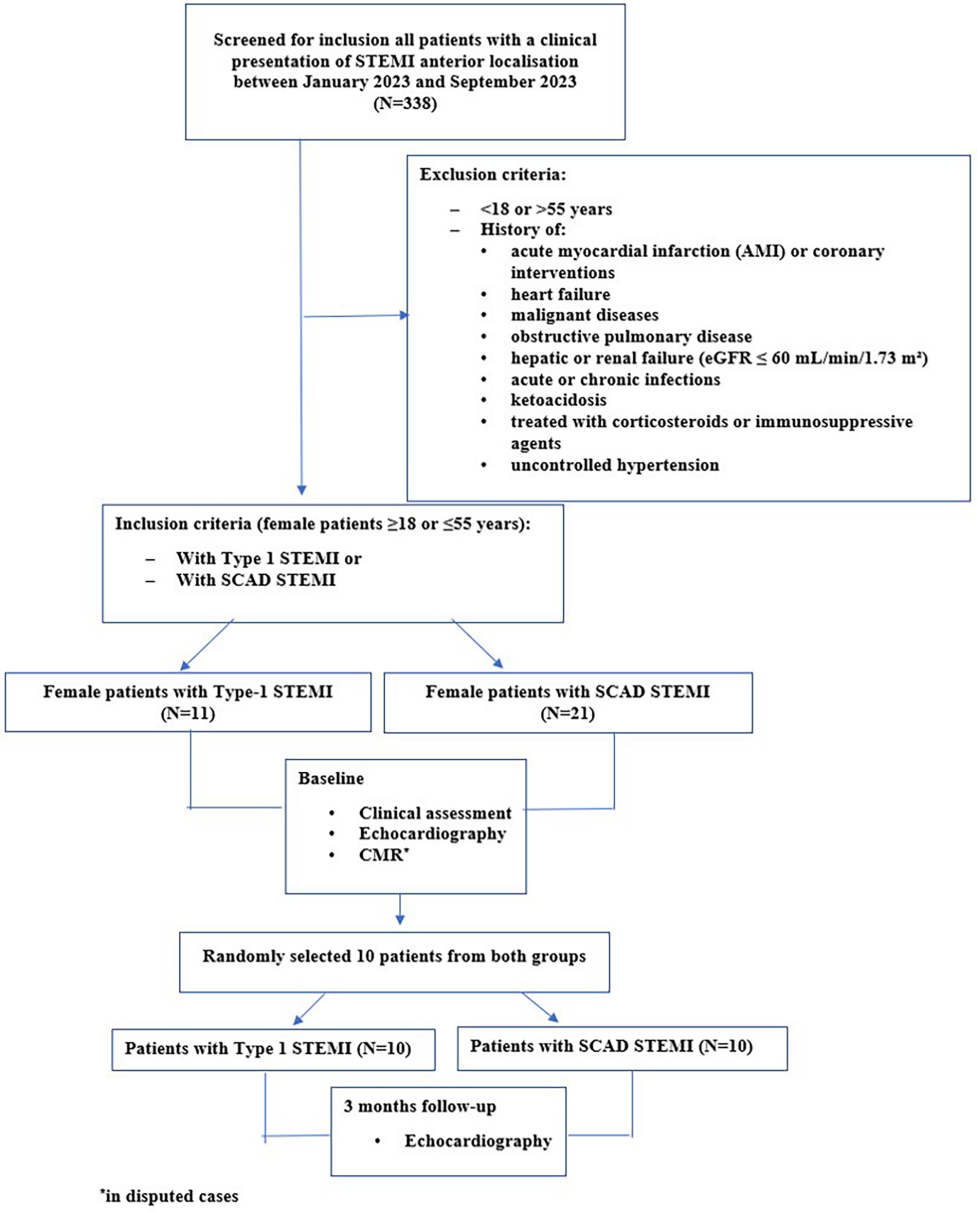
Flowchart of the study.

Two to four weeks after the initial measurements, an echocardiographic exam, including strain analysis, was repeated in 10 randomly selected patients from both groups (SCAD-STEMI and type 1 STEMI) by the same observer (G.K.). The flow chart of the study is presented in Figure 1.

### 2.1 Statistical analyses

The continuous variables are presented as mean ± SD, while categorical data are presented as percentages. The differences between the groups at baseline were tested using a one-way analysis of variance (ANOVA), while the χ^2^ test was used for categorical variables. We analyzed differences in 3-month follow-up echocardiographic parameters in 10 randomly selected patients from both groups (SCAD-STEMI and type 1 STEMI) by using the Student *t*-test. The statistical analyses were performed using SPSS software version 20.0 (SPSS Inc., USA) with a significance level set at *p* < 0.05.

## 3 Results

We analyzed two groups of female patients ≤55 years of age, presenting with either SCAD-STEMI due to LAD dissection or type 1 STEMI due to atherothrombotic LAD occlusion. As presented in Table 1, the two groups were similar with respect to age, risk factors, and comorbidities. However, patients with SCAD-STEMI had higher systolic and lower diastolic blood pressures and higher heart rates compared with type 1 STEMI patients.

**Table 1:** Baseline clinical and laboratory characteristics, risk factors, and therapeutic approaches in SCAD-STEMI and type 1 STEMI groups of patients.

	SCAD-STEMI (*n* = 11)	Type 1 STEMI (*n* = 21)	*p*
Age, years (mean ± SD)	45.1 ± 7.3	46.2 ± 6.7	0.671
BMI, kg/m^2^ (mean ± SD)	24.8 ± 4.2	26.1 ± 4.1	0.516
Hypertension (%)	47.4	66.7	0.339
Hyperlipidemia (%)	33.3	22.2	0.535
Diabetes (%)	0	0	1.000
Renal insufficiency (%)	0	0	1.000
Family history of coronary diseases (%)	0	22.2	0.125
Smoking (%)	33.3	66.7	0.100
Stressful situation (%)	27.3	23.9	0.830
Pregnancies/postpartum (%)	18.2	4.8	0.216
Systolic BP, mmHg (mean ± SD)	147.5 ± 32.2	122.2 ± 23.5	**0.049**
Diastolic BP, mmHg (mean ± SD)	67.3 ± 19.4	73.3 ± 9.1	**0.022**
Heart rate, bpm (mean ± SD)	82.5 ± 14.1	71.5 ± 8.1	**0.027**
SCAD type (%)	/	/	
1	18.2		
2	36.3		
3	27.3		
4	18.2		
TIMI (0/1/2/3) (%)			**0.012**
0	27.3	81.0	
1	18.1	9.5	
2	27.3	9.5	
3	27.3	0	
Localization of occlusion, *n* (%)			0.420
LAD	11 (100)	18 (85.7)	
LAD + Cx	0	2 (9.5)	
LAD + D1	0	1 (4.8)	
Time from symptom onset to PCI center admission, h (mean ± SD)	2.97 ± 2.06	2.60 ± 2.70	0.499
Heart failure (Killip class ≥ 2) (%)	22.2	10.5	0.409
Arrhythmia (%)			
Ventricular tachycardia	22.2	47.6	0.193
Ventricular fibrillation	12.5	19.0	0.677
Atrial fibrillation	0	5.3	0.600
High-sensitivity troponin T (ng/L)	1,407.7 ± 410.9	1,833.7 ± 600.0	0.716
Creatine kinase (U/L)	867.7 ± 206.1	1,894.4 ± 468.0	0.208
NT-pro BNP (pg/ml)	174.6 ± 123.5	1,401.4 ± 700.7	0.187
Total cholesterol (mmol/L)	4.6 ± 0.7	4.9 ± 1.6	0.730
High-density lipids (mmol/L)	1.7 ± 0.4	1.2 ± 0.4	**0.029**
Low-density lipids (mmol/L)	2.4 ± 0.5	3.2 ± 1.1	0.163
Triglyceride (mmol/L)	1.2 ± 0.4	1.1 ± 0.5	0.735
hs-CRP (ng/L)	55.9 ± 33.1	27.5 ± 14.8	0.374
Aspirin (%)	81.8	90.5	0.482
P2Y12 inhibitors (%)			
Clopidogrel	72.7	33.3	**0.006**
Ticagrelor	0	57.2	
Anticoagulant therapy (%)	72.7	90.5	0.135
ACE inhibitors (%)	45.5	81.0	**0.040**
BB (%)	54.5	85.7	0.053
Statins (%)	45.5	90.5	**0.005**
PCI (stent/POBA) (%)	36.4	100	**0.001**

BB, beta-blockers; BMI, body mass index; BP, blood pressure; Cx, circumflex coronary artery; D1, first diagonal branch coronary artery; NT-pro BNP, N-terminal pro-B-type natriuretic peptide; POBA, balloon angioplasty without a stent.

Bold values denote significant differences (*p* < 0.05).

There were no differences in the levels of high-sensitive troponin T (hs-troponin T), N-terminal pro-brain natriuretic peptide (NT-proBNP), and high-sensitive C reactive protein (hs-CRP) (Table 1). The frequency of symptomatic heart failure and arrhythmias occurring during the acute phase was similar between the groups. SCAD-STEMI patients more often had TIMI 3 flow at angiography as opposed to patients with type 1 STEMI, who more often had TIMI 0 flow. Furthermore, the use of reperfusion strategy with primary PCI was more frequent in patients with type 1 STEMI compared to SCAD-STEMI, as well as the use of dual antiplatelet therapy.

The results of the echocardiographic and CMR assessment of LV function at baseline of SCAD-STEMI and type 1 STEMI patients are presented in Table 2. Between-group comparisons at baseline showed no significant differences in clinical, echocardiographic, and CMR parameters, including infarct size at baseline, as assessed by the extent of LGE. The only observed difference at baseline was a higher LV mass index assessed by echocardiography in patients with type 1 STEMI (Table 2).

**Table 2:** Differences in echocardiographic and CMR parameters between SCAD-STEMI and type 1 STEMI patients at baseline.

Baseline echocardiography	SCAD-STEMI (*n* = 11)	STEMI type 1 (*n* = 21)	*p*
LVEDD (cm)	4.9 ± 0.4	5.0 ± 0.6	0.968
LVESD (cm)	3.2 ± 0.4	3.2 ± 0.8	0.879
LVIVS (cm)	0.98 ± 0.17	0.98 ± 0.19	0.155
LVPW (cm)	0.87 ± 0.15	0.95 ± 0.14	0.137
LV mass index (g/m^2^)	72.2 ± 14.0	97.6 ± 21.4	**0.019**
WMI	1.48 ± 0.43	1.51 ± 0.43	0.859
Peak E wave velocity (m/s)	0.66 ± 0.16	0.58 ± 0.17	0.255
Peak A wave velocity (m/s)	0.62 ± 0.15	0.62 ± 0.12	0.993
E/A ratio	1.10 ± 0.44	0.91 ± 0.42	0.277
Peak e′ medial velocity (cm/s)	6.5 ± 1.3	7.7 ± 3.0	0.433
Peak e′ lateral velocity (cm/s)	8.5 ± 1.9	8.5 ± 3.1	0.989
E/e′ average ratio	7.7 ± 2.6	7.8 ± 1.6	0.958
LAV (ml)	40.5 ± 7.6	32.5 ± 12.8	0.242
LAVI (ml/m^2^)	23.2 ± 5.1	18.2 ± 6.7	0.168
LVEDV (ml)	122.3 ± 18.3	101.5 ± 39.8	0.112
LVEDVI (ml/m^2^)	60.0 ± 16.6	54.3 ± 31.4	0.073
LVESV (ml)	69.5 ± 8.3	56.5 ± 19.8	0.579
LVESVI (ml/m^2^)	33.7 ± 9.2	30.2 ± 16.1	0.511
LVEF (%)	48.0 ± 7.1	48.6 ± 11.4	0.881
LVGLS (%)	−14.0 ± 2.77	−13.3 ± 4.5	0.630
Baseline CMR	SCAD-STEMI (*n* = 10)	STEMI type 1 (*n* = 10)	*p*
LVEDV (ml)	144.2 ± 36.5	151.8 ± 28.2	0.789
LVEDVI (ml/m^2^)	83.3 ± 17.8	82.4 ± 10.8	0.948
LVESV (ml)	72.1 ± 33.8	75.7 ± 26.1	0.892
LVESVI (ml/m^2^)	41.3 ± 18.1	42.0 ± 13.7	0.958
LVEF (%)	50.8 ± 11.1	43.3 ± 13.3	0.495
LGE (%)	10.0 ± 8.8	14.3 ± 6.6	0.536

LAV, left atrial velocity; LAVI, left atrial velocity index; LVEDD, left ventricular end-diastolic dimension; LVESD, left ventricular end-systolic dimension; LVIVS, left ventricular interventricular septum dimension; LVPW, left ventricle posterior wall dimension; WMI, wall motion index.

Bold values denote significant differences (*p* < 0.05).

The results of comparisons in echocardiographic parameters between 10 randomly selected patients (from both groups) from baseline to the 3-month follow-up are presented in Table 3. There was a tendency toward a decrease in left ventricular end-diastolic volume (LVEDV)/left ventricular end-diastolic volume index (LVEDVI) and left ventricular end-systolic volume (LVESV)/left ventricular end-systolic volume index (LVESVI) in SCAD-STEMI patients, whereas there was a tendency toward an increase in LVEDV/LVEDVI and LVESV/LVESVI in type 1 STEMI patients (Table 3). However, the difference in LV volumes from baseline to 3-month follow-up was not statistically significant between the two groups (Table 3). There was a significant difference in left ventricular ejection fraction (LVEF) between the two groups, driven by the numerically greater improvement in LVEF in SCAD-STEMI patients than in type 1-STEMI patients (Table 3). Left ventricular global longitudinal strain (LVGLS) was not statistically different at follow-up between the two groups (Table 3).

**Table 3:** Improvements of echocardiographic parameters after 3-month follow-up in SCAD-STEMI patients and type 1 STEMI patients.

Δ (3-month FU—baseline)	SCAD-STEMI (*n* = 10)	Type 1 STEMI (*n* = 10)	*p*
Δ LVEDV, (LVEDV_2_ − LVEDV_1_) (ml)	−11.6 ± 21.9 (107.2 ± 32.6)—(118.9 ± 18.9)	8.4 ± 28.3 (125.2 ± 40.7)—(117.2 ± 52.9)	0.122
Δ LVEDVI, (LVEDVI_2_ − LVEDVI_1_) (ml/m^2^)	−10.9 ± 13.7 (57.3 ± 17.1)—(68.1 ± 8.3)	3.9 ± 15.3 (67.7 ± 18.1)—(63.8 ± 26.1)	0.055
Δ LVESV, (LVESV_2_ − LVESV_1_) (ml)	−11.8 ± 16.3 (49.6 ± 17.3)—(61.5 ± 19.5)	1.1 ± 22.6 (64.0 ± 33.9)—(62.9 ± 44.4)	0.200
Δ LVESVI, (LVESVI_2_ − LVESVI_1_) (ml/m^2^)	−7.8 ± 9.7 (26.8 ± 9.6)—(34.6 ± 10.7)	0.3 ± 11.8 (34.2 ± 16.5)—(33.9 ± 22.5)	0.150
Δ LVEF, (LVEF_2_ − LVEF_1_) (%)	10.1 ± 5.3 (57.7 ± 7.2)—(47.6 ± 7.3)	1.8 ± 5.1 (52.6 ± 11.3)—(50.8 ± 11.8)	**0** **.** **002**
Δ LVGLS, (LVGLS_2_ − LVGLS_1_) (%)	−4.6 ± 2.9 (−18.1 ± 3.9)—(−13.4 ± 2.1)	−2.0 ± 2.8 (−16.2 ± 6.5)—(−14.2 ± 4.9)	0.055

FU, follow-up; LVEDV_1_, left ventricular end-diastolic volume at baseline; LVEDV_2_, left ventricular end-diastolic volume after 3-month FU; LVEDVI_1_, left ventricular end-diastolic volume index at baseline; LVEDVI_2_, left ventricular end-diastolic volume index after 3-month FU; LVEF_1_, left ventricular ejection fraction at baseline; LVEF_2_, left ventricular ejection fraction after 3-month FU; LVESV_1_, left ventricular end-systolic volume at baseline; LVESV_2_, left ventricular end-systolic volume after 3-month FU; LVESVI_1_, left ventricular end-systolic volume index at baseline; LVESVI_2_, left ventricular end-systolic volume index after 3-month FU; LVGLS_1_, left ventricular global longitudinal strain at baseline; LVGLS_2_, left ventricular global longitudinal strain after 3-month FU.

Bold values denote significant differences (*p* < 0.05).

**Figure 2:**
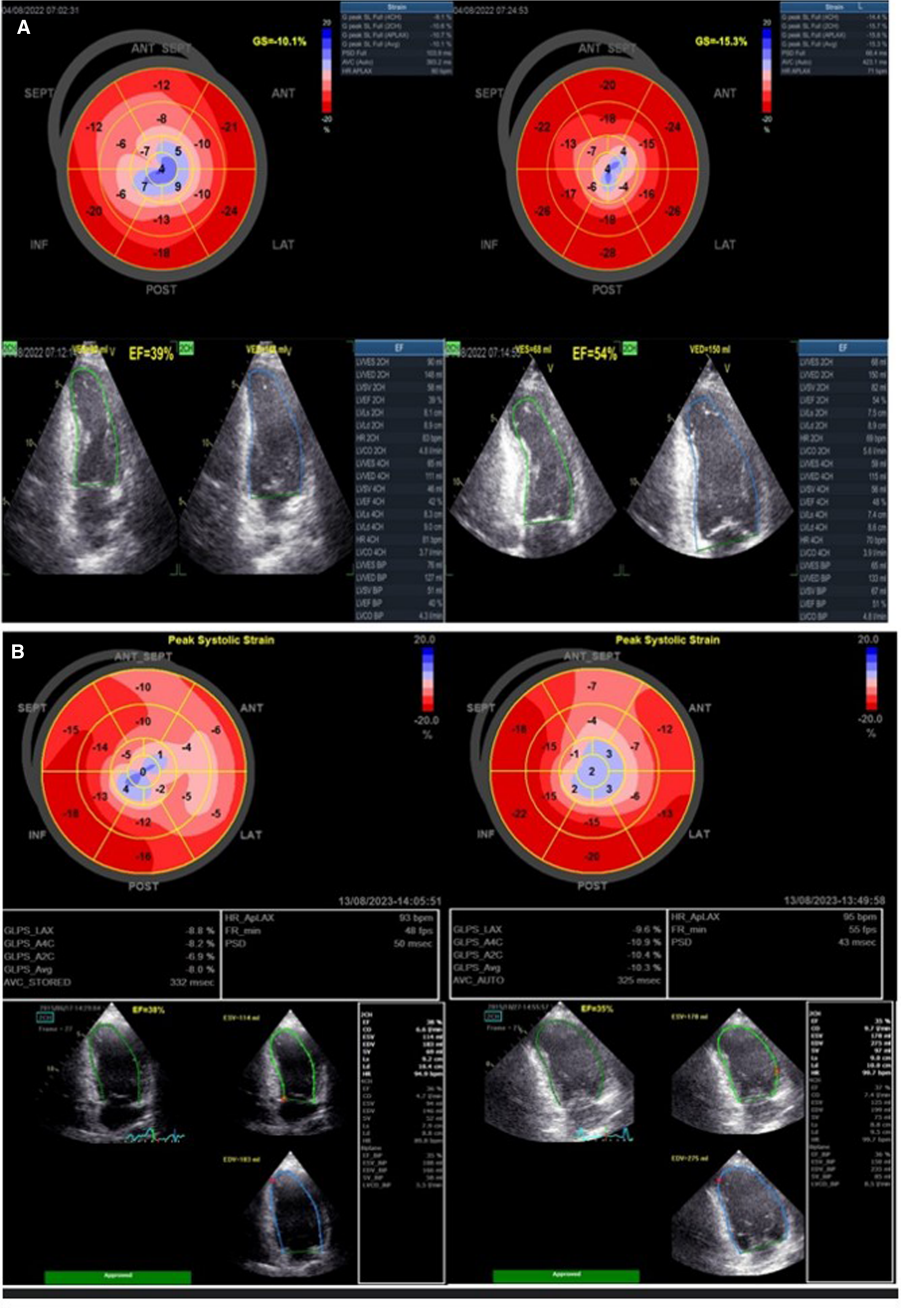
(**A**) A 42-year-old woman presented with STEMI anterior localization SCAD on left anterior artery type 4 and TIMI flow 0, treated with percutaneous transluminal coronary angioplasty (PTCA) without implantation of stents. Baseline LVEF was 39% and LVGLS was −10.1%. After 3-month follow-up, the LVEF was 54% and LVGLS was −15.3%. (**B**) A 29-year-old woman presented with type 1 STEMI anterior localization due to occluded LAD treated with primary PCI and implantation of two stents. Baseline LVEF was 35% and LVGLS was −8.0%. After 3-month follow-up, the LVEF was 36% and LVGLS was −10.3%.

Figure 2 illustrates the changes in myocardial function (LVEF and LVGLS) in two female patients, one with SCAD-STEMI and the other with type 1 STEMI, assessed from baseline to the 3-month follow-up.

In the Supplementary Material, we also illustrate CMR differences in the distribution of LGE at baseline in a type 1 STEMI patient and a SCAD-STEMI patient (Supplementary Figure S1) as well as in other CMR findings (Supplementary Figures S2, S3).

## 4 Discussion

In this prospective study, we compared female patients with SCAD-STEMI and type 1 STEMI with culprit LAD by analyzing their clinical features and imaging findings at baseline and the 3-month follow-up. Our results suggest that patients with SCAD-STEMI, despite having similar baseline clinical characteristics, estimates of infarct size (LGE), and LV function to type 1 STEMI patients, might have a more favorable trajectory of LV remodeling over the 3-month follow-up. Although the observed differences in LV volumes between the two patient groups were not significant at 3-month follow-up, patients with SCAD-STEMI experienced a net decrease in LV volumes, which was not observed in type 1 STEMI patients. There was a significant difference in LVEF at 3-month follow-up between the two groups due to a greater net improvement in LVEF in SCAD-STEMI patients compared with the type 1 STEMI group. LVGLS was not significantly different, albeit both groups showed signs of some improvement in myocardial strain at 3-month follow-up.

Previous studies suggested that clinical, electrocardiographic, and echocardiographic findings may be similar in SCAD-STEMI and type 1 STEMI patients, which may carry a risk of an inaccurate diagnosis or inadequate treatment if the two conditions are not differentiated (10, 11). It is also important to consider that clinical presentation of both SCAD-STEMI and type 1 STEMI can vary widely among individuals, and infarct size might not always follow a clear pattern based solely on the pathophysiology of myocardial infarction (5). Of note, SCAD is a condition that occurs more frequently in women and is the prevailing cause of myocardial infarction in young and middle-aged females without cardiovascular risk factors (1). It is often precipitated by stressful situations, strenuous exercise, hormonal changes, pregnancy, vasospasm, connective tissue disorders, fibromuscular dysplasia, and the use of certain medications or drug abuse (cocaine) (1, 3). In addition, depression has been described as a risk factor not only associated with a higher risk of SCAD but also with the development and progression of atherosclerosis, potentially leading to type 1 myocardial infarction (12).

Although our study had a small sample size for the two groups of females ≤55 years old, it is still informative to note that the two groups were well-balanced in baseline characteristics, risk factors, laboratory analyses, and immediate clinical course. However, there were significant differences in TIMI flow on angiography (i.e., type 1 STEMI patients more often had TIMI 0 flow) and reperfusion strategy treatment with primary PCI, and the use of dual antiplatelet therapy was more frequent in patients with type 1 STEMI.

### 4.1 Pathophysiological characteristics in SCAD-STEMI

The pathophysiological mechanisms underlying SCAD-STEMI and type 1 STEMI conditions differ, which may result in differences in infarct size and post-infarction LV remodeling. Although an intimal tear represents the most frequent cause of SCAD, causing a formation of a false lumen in the medial layer, coronary intramural hematoma without an intimal tear was also documented with the use of intravascular ultrasonography (10,11, 13) and later confirmed by high-resolution optical coherence tomography (14). The primary cause of an AMI in SCAD is the obstruction of a coronary artery due to either the compression of the artery's true lumen by a dissection flap or the expansion of a hematoma within the arterial wall. However, subsequent SCAD healing and a conditioning effect on the myocardium by coronary artery collateralization induced by prior fixed stenosis (similar to type 1 STEMI) may have an impact on the infarct size in SCAD-STEMI (5). These explanations point to a dynamic interplay of mechanisms affecting the infarct size in SCAD-STEMI (5).

### 4.2 SCAD type by angiography and formation of myocardial infarction

The SCAD type by angiography may also have an impact on the formation of myocardial infarct size. In our study, 18.2% of patients had SCAD type 1; 36.3% of patients had SCAD type 2; 27.3% SCAD type 3; and 18.2% of patients who went directly to PCI and revascularization had SCAD type 4. In SCAD type 1, the longitudinal filling defect can be detected due to the formation of an intimal flap (15). SCAD type 2 (the most common presentation) is characterized by a diffuse, long, smooth tubular stenosis caused by intramural hematoma without an apparent dissection (15). Type 1 and 2 SCAD patients may result in smaller myocardial infarction size, which may explain the more favorable resolution of myocardial infarct size and improvement in parameters of LV function in SCAD-STEMI patients in our study. Most experts believe that intramural hematoma is the initial mechanism in most SCAD and that there would be some time interval between intramural hematoma generation (type 2 lesion) and the development of a type 1 lesion (16). This concept may explain findings that type 1 lesions were more frequently found in “late presenters” in whom SCAD lesions had more time to produce myocardial ischemia and/or necrosis (16). In addition, myocardial infarction size further depends on the characteristics of coronary vessel involvement, with larger infarctions caused by the proximal, multi-segment and/or multivessel SCAD (5). SCAD type 3 can occur due to focal or multiple tubular lesions, usually <20 mm long, caused by intramural hematoma that can mimic atherosclerosis and require intravascular imaging for diagnosis (15). The increase in the severity of coronary artery stenosis and the presence of fenestrated and non-fenestrated types of stenosis can also influence the infarct size (14). SCAD type 4 has been described as a complete vessel occlusion (17). Patients with SCAD type 4 exhibit similarities with type 1 STEMI patients in myocardial infarct size, and it seems that these patients have larger myocardial infarctions than SCAD type 1 and 2 patients. In patients with SCAD associated with poor TIMI flow, who are at an increased risk of developing a larger infarct size, the therapeutic strategy may favor interventional management over conservative treatment (17).

### 4.3 Imaging methods for quantifying myocardial infarct size and myocardial perfusion in SCAD-STEMI due to LAD dissection and type 1 STEMI due to LAD occlusion

Myocardial infarct size can be quantified with a high degree of precision using CMR imaging by a semi-automatic method with LGE (18). CMR-quantified infarct size can be categorized as large (LGE mass accounting for >10% of the total LV mass) or small (LGE mass accounts for ≤10% of the total LV mass) (5). In the case of SCAD-STEMI patients, earlier research demonstrated a trend toward smaller myocardial infarct size and reduced levels of LGE with both endocardial and transmural involvement, in comparison to the type 1 STEMI patients where the characteristic pattern of LGE involved subendocardial distribution (19). However, in our study, baseline CMR-LGE values were not significantly different between the two groups.

Besides CMR, stress-test perfusion CMR, single-photon emission computed tomography or positron emission tomography myocardial perfusion imaging, and intravascular Doppler ultrasound coronary flow reserve can be used to further assess myocardial infarct size and blood flow and identify areas of reduced myocardial perfusion caused by SCAD (20–23). The imaging modalities are particularly valuable in assessing coronary microvascular dysfunction, however, with caveats imposed by limited availability and optimal timing of the assessment following a SCAD event. Stress perfusion tests are contraindicated in the acute phase of the disease, but they can be useful later in the follow-up of SCAD patients. Reassessment of cardiac function at 3 months is appropriate for patients with reduced LV function at the time of an AMI (24). Further limitations include breast or soft tissue attenuation and reduced accuracy in patients with smaller-sized hearts, which are more commonly seen in women than men (25).

### 4.4 Imaging methods for the assessment of myocardial function in SCAD-STEMI due to LAD dissection and type 1 STEMI due to atherothrombotic LAD occlusion

CMR is regarded as a standard reference method for the assessment LV myocardial function after an AMI (26). Cine-imaging CMR has been previously used to determine LV volumes and global and regional function at baseline and follow-up (5). Echocardiography is a more available but less accurate method for the assessment of LV function compared with CMR. The study by Franco et al. (27) suggested that approximately 26% of SCAD patients had a slightly reduced LVEF below 50% and approximately 5.1% had an LVEF below 40%. In the Spanish Registry of SCAD patients (SR-SCAD), patients with SCAD and reduced LVEF <50% presented more often with an anterior STEMI and multi-segment involvement coronary artery disease (16). These findings are in line with our observations. In the present study, the mean values of LVEF at the baseline were below 50% in both groups (48.0% vs. 48.6%, SCAD-STEMI vs. STEMI type 1). At 3-month follow-up, we found that the mean values had increased to >50% in both groups (57.7 vs. 52.6%, SCAD-STEMI vs. type 1 STEMI). We used LVGLS in this study because it can provide more precise prognostic information in SCAD survivors, particularly those with an LVEF > 50%, compared with other imaging options (28, 29). Although there were no statistically significant differences between the two groups both at baseline and 3-month follow-up in LVGLS, some improvement was observed in LVGLS in both groups over time. It remains to be determined whether strain echocardiography can add to the monitoring of patients with SCAD, considering the limited availability of those other diagnostic methods, such as CMR, in everyday practice.

A previous position paper recommended that SCAD patients who are experiencing recurrent chest pain should be carefully assessed via serial electrocardiography (ECG), high-sensitivity troponin measurement, and coronary angiography imaging in accordance with the physician's assessment (15). Therefore, the significance of the assessment of LV systolic function and myocardial infarct size is high and mandatory to guide further pharmacological and non-pharmacological management. First, early CMR imaging in SCAD-STEMI patients may provide identification of high-risk markers for future adverse cardiac events. Second, there is a need for continued and extended monitoring of SCAD-STEMI patients beyond 3 months to enable a more comprehensive assessment of their cardiac function and identification of long-term complications, including the development of heart failure (HF).

## 5 Study limitations

Several limitations of the present study need to be acknowledged. The most important limitation is a small sample size; however, the study was prospective and multicenter, which mitigates the limitation imposed son the generalizability of our findings. Furthermore, we only analyzed female patients ≤55 years old, which limits generalization to older women or men. Another limitation is that we did not perform CMR in all patients at baseline, which imposes a caveat in the interpretation of CMR estimated infarct sizes (extent of LGE) between the two groups. The study is also limited by short follow-up time. However, using one of the more sophisticated echocardiographic imaging methods, we managed to find a difference in myocardial function between the two observed groups. Considering the limitations of our study, its findings should be regarded as hypothesis generating, pending further confirmation from larger analyses.

## 6 Conclusions

The results of the present study suggest that young and middle-aged female patients with SCAD-STEMI exhibit a tendency for an improvement in LV systolic function during the prospective follow-up, which was more substantial in comparison to patients with type 1 STEMI. These differences may be related to a greater prevalence of TIMI 3 flow at angiography in SCAD-STEMI patients, subsequent healing of the dissected artery, and an overall smaller ischemic burden in SCAD-STEMI compared with type 1 STEMI patients. However, this may not be the case with the more complex types of SCAD, involving total vessel occlusion and multisegmented or multivessel engagement. Multimodality imaging, such as standard and strain echocardiography and CMR, may play a valuable role in the initial evaluation and follow-up of patients with SCAD-STEMI and in the assessment of the trajectory of LV remodeling following SCAD-STEMI, which may have important therapeutic and prognostic implications.

## Supplementary material

SUPPLEMENTARY FIGURE S1

Two middle age females with STEMI presentation on CMR. Panel A and C presented a short axis and 4-chamber view of the left ventricle (LV) of female 48 years old with type-1 STEMI and occluded LAD treated with primary percutaneous coronary intervention (PCI) and implantation 3 stents in LAD. Panel B and D presented a short axis and 4-chamber views of LV of a female 42 years old presented with SCAD-STEMI due to type 4 LAD dissection treated with percutaneous balloon dilatation without stent implantation.

SUPPLEMENTARY FIGURE S2

Additional CMR findings of the female patients in Figure 3.

SUPPLEMENTARY FIGURE S3

Female with SCAD type 2 and TIMI 3 with preserved LVEF, without abnormal contractility, and without CMR LGE verification.
