# Supplementary figures and images for: Differences in left ventricular myocardial function and infarct size in female patients with ST elevation myocardial infarction and spontaneous coronary artery dissection
Source: Front Cardiovasc Med. 2024 Jan 8;10:1280605. doi: 10.3389/fcvm.2023.1280605 (PMC10800883; doi:10.3389/fcvm.2023.1280605)

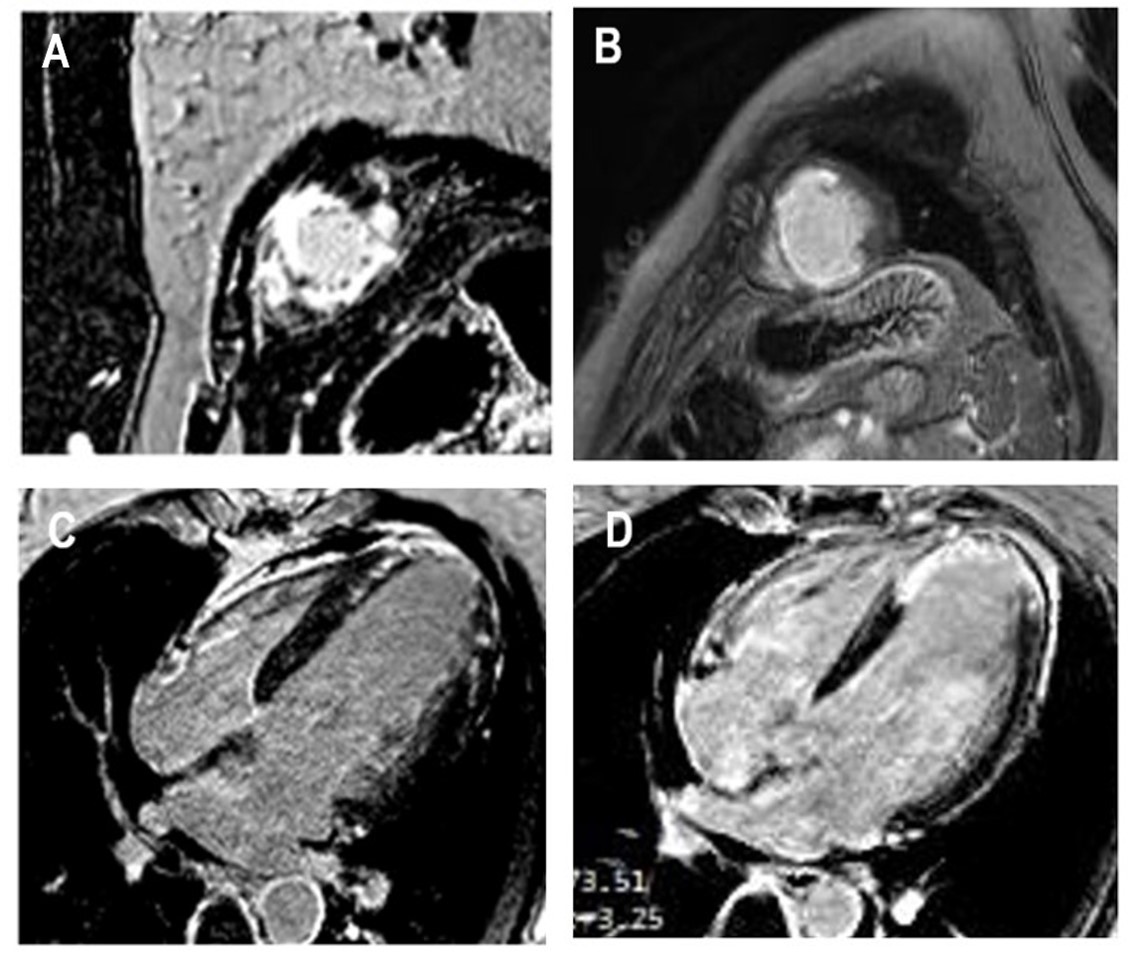

Supplement: Supplementary file 1 [file Image1.jpeg]

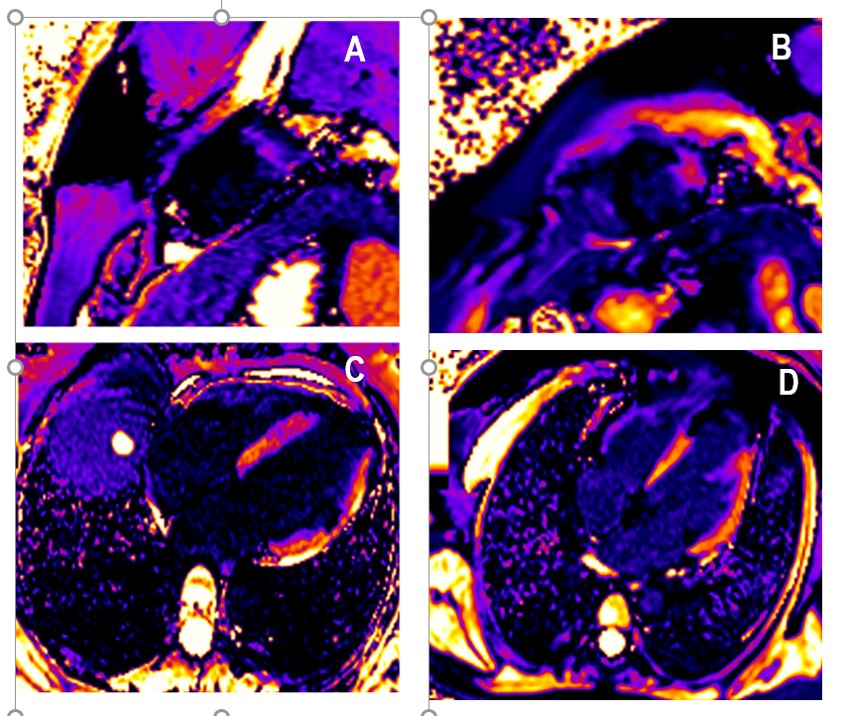

Supplement: Supplementary file 2 [file Image2.jpeg]

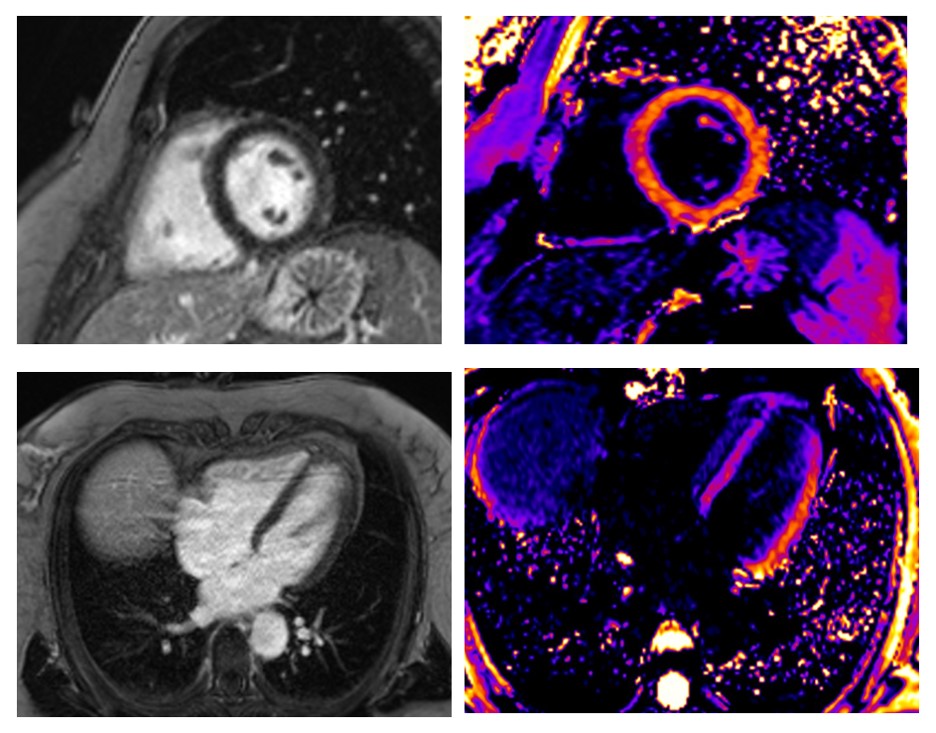

Supplement: Supplementary file 3 [file Image3.jpeg]
